# Comprehensive and Conservative Management of Talon Cusp: A New Technique

**DOI:** 10.1155/2016/5843231

**Published:** 2016-12-26

**Authors:** Ankit Arora, Padmaja Sharma, Surendra Lodha

**Affiliations:** ^1^Department of Conservative Dentistry and Endodontics, M. P. Dental College, Hospital and Oral Research Institute, Vishwajyoti Ashram, Munjmahuda, Vadodara, Gujarat 390011, India; ^2^Department of Orthodontics and Dentofacial Orthopaedics, M. P. Dental College, Hospital and Oral Research Institute, Vishwajyoti Ashram, Munjmahuda, Vadodara, Gujarat 390011, India; ^3^Department of Orthodontics and Dentofacial Orthopaedics, Jaipur Dental College, Jaipur, Rajasthan, India

## Abstract

Talon cusp is a common dental anomaly affecting maxillary central incisors. Gradual grinding of this additional cusp is commonly followed now. Advocated below is a new technique explaining the use of air abrasion and putty index during the stepwise reduction of the cusp. The technique is advantageous in preventing patient discomfort and tracking the amount of reduction in a predictable way.

## 1. Introduction

Talons cusp is a common developmental anomaly of permanent dentition [1]. The anomalous cusp is composed of enamel, dentin, and pulpal tissue with varying extension [2]. Mellor and Ripa in 1970 coined the term talon cusp because of its resemblance to an eagle's talon [3]. Hattab et al. classified the anomalous cusp based on the degree of its formation and extension [1]. Talon's cusp frequently interferes with the orthodontic tooth movement of lateral incisors during their retraction. This leads to premature contact, occlusal trauma, and reversible apical periodontitis of the opposing tooth [2]. The developmental grooves present between the Talons cusp and the palatal surface of the tooth can harbor microorganisms and lead to future caries. This anomaly demands early and preventive intervention especially where orthodontic movement of the tooth is anticipated [4].

Talons cusp treatment mainly involves reduction of the cusp and management of the developmental grooves associated with it. Gradual grinding of the cusp and use of air abrasion allow us to follow the principles of minimally invasive dentistry in treating this anomaly. Explained here is a new technique to conservatively manage the anomaly in a more predictable way while reducing the discomfort to the patient.

## 2. Case Report

**Figure 1:**
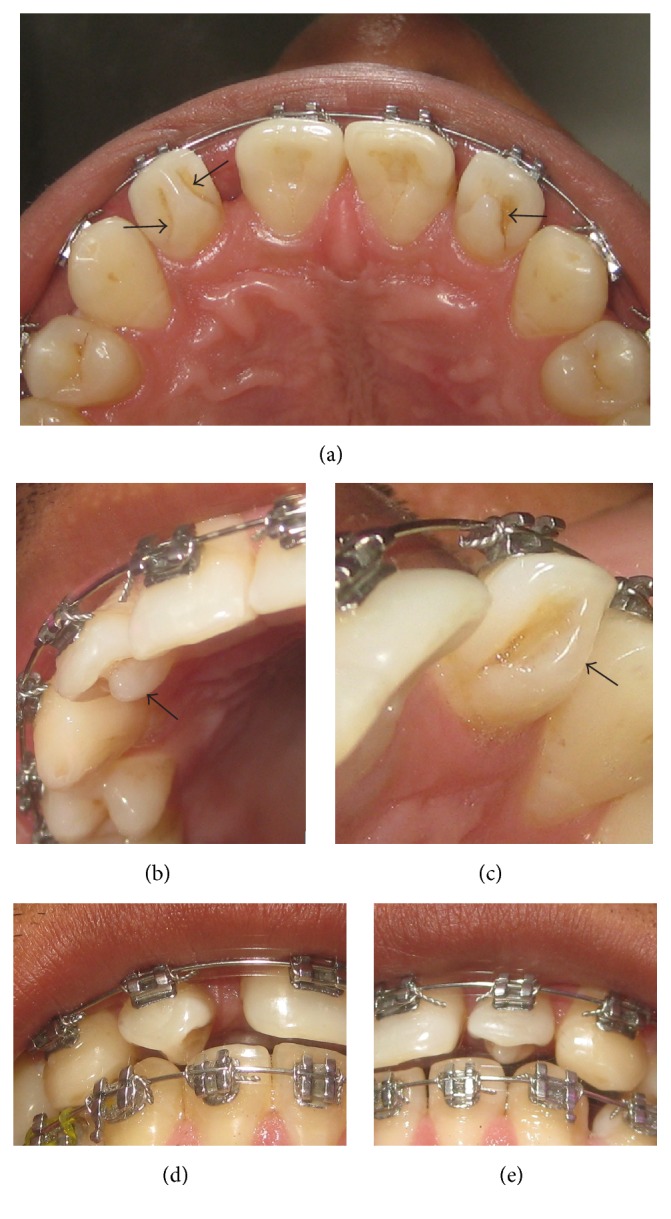
(a–c) Presence of complete Talon cusp (black arrows); (d, e) incisal view showing the contact of Talon cusp with the lower anterior teeth.

A 23-year-old male patient undergoing orthodontic treatment was referred to the Department of Operative Dentistry and Endodontics for the treatment of bilateral Talon's cusp on maxillary lateral incisors. Elimination of Talon's cusp was deemed necessary to allow retraction of upper anterior teeth. Both lateral incisors presented additional cusp on the palatal surface extending up to the incisal edge (Figures 1(a)–1(c)). Deep stained developmental grooves were observed at the junction of the talon and the palatal surface of the tooth (Figure 1(a)). In occlusion, the talons were almost in contact with the lower anterior teeth (Figures 1(d) and 1(e)). Hence a diagnosis of Type 1 Talon was formulated and conservative method of treatment involving gradual grinding of the additional cusp was planned.

**Figure 2:**
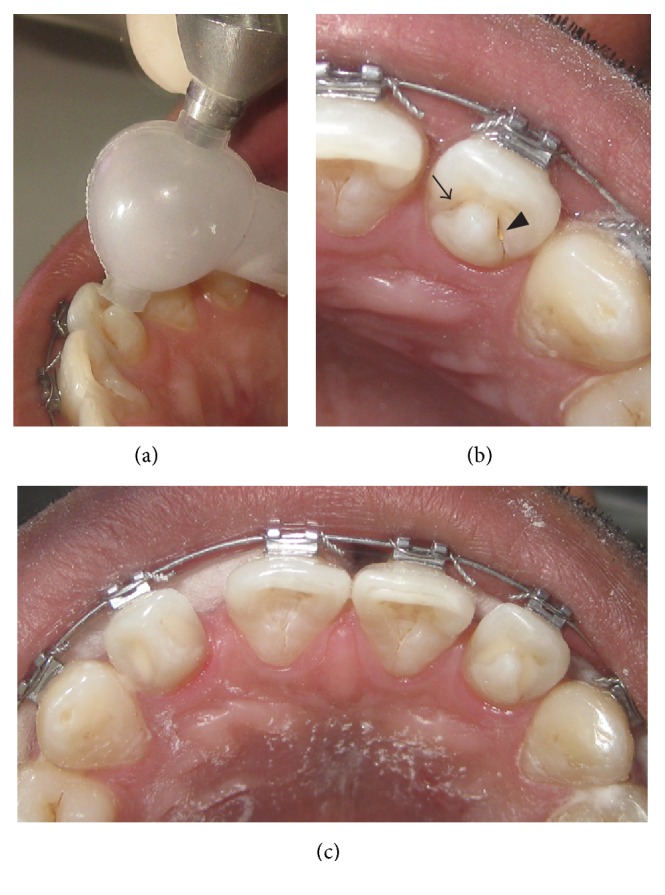
(a) Use of sand trap while using air abrasion; (b) prepared groove (long arrow) and unprepared groove (short arrow); (c) sealing of the grooves with GIC.

It was planned to seal the deep developmental grooves with glass ionomer cement till the time they were eradicated in the process of grinding. Air abrasion was used for minimal preparations of the grooves (Figures 2(a) and 2(b)). 50 *μ*m alumina particles were used in Microetcher ERC (Danville materials, San Ramon, CA) at 60 psi for 3 seconds at a stretch. To avoid spilling of alumina particles inside oral cavity and to keep the stream focused on to the target site, a sand trap was used (Figure 2(a)). At the end of the preparation, the discolored groove was clean and wide enough to receive the restorative material (Figure 2(b)). Glass ionomer cement (GIC) was used to restore the cavity (Figure 2(c)).

**Figure 3:**
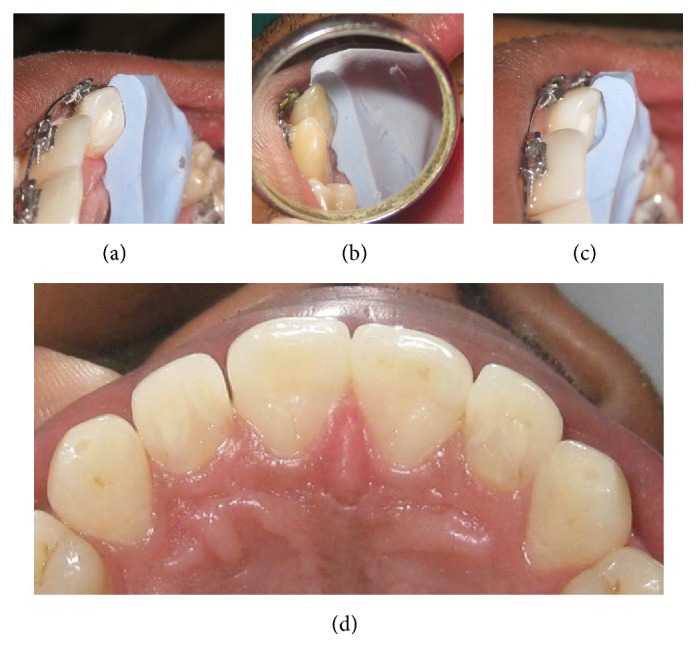
(a–c) Putty index used for tracking the amount of reduction; (d) following completion of treatment.

In the next step, before starting the grinding of the cusp a putty index was made and cut sagittally at the maximum height of Talon's cusp for both lateral incisors. Hence adaptation of putty index to Talon's cusp was visible (Figure 3(a)). The objective was to track the reduction of the cusp in every appointment. Subsequently, in 3 visits separated by 4-week interval, grinding was carried out on the lateral side of Talon's cusp to eliminate it. In each visit, the putty index guided the amount of reduction which was limited to 1 mm (Figures 3(b) and 3(c)). Patient was advised to use desensitizing tooth paste during this time period. Patient experienced only mild sensitivity during grinding of the cusp or during the intervals. Complete grinding of the cusp allowed adequate space for orthodontic retraction of the teeth. At the end of the orthodontic treatment, patient was found to be completely asymptomatic and the teeth were in alignment with spaces closed (Figure 3(d)).

## 3. Discussion

The additional cusp on the palatal surface was diagnosed as Type 1 Talon because it extended to more than half the distance from the cementoenamel junction to the incisal edge [1]. There are three important aspects linked with the conservative treatment of Talons cusp: (i) treating the deep developmental grooves present at the junction of additional cusp and palatal surface of the tooth, (ii) the site of reduction, and (iii) amount of reduction of Talon's cusp. The technique explained above is entirely based on the principles of minimal intervention dentistry as it allows a very precise and limited removal of dental tissues preserving the natural tissue [5].

It is advocated to seal the deep developmental grooves to prevent caries [1]. Hence, they were cleaned and conservatively prepared by using air abrasion and sealed with GIC. Air abrasion allows ultraconservative preparations with minimal patient discomfort and apprehension. Apart from reduced noise, vibration, and sensitivity associated with air abrasion, it is also very effective, efficient, and precise [6, 7].

Stepwise or gradual reduction of Talon's cusp was followed as it preserves the pulp vitality and averts the risk of pulp exposure and discomfort associated with deep dentinal preparation [4, 8]. The grinding was carried out along the side of the cusp and not the tip of the cusp because most of the odontoblasts lie along the length of the cusp. These odontoblasts promote deposition of reparative dentin over a period of few weeks [9, 10]. The putty index contributed as a reference tool. It allowed tracking the amount of reduction which was to be done in each visit and it also guided in grinding the cusp evenly along the side of the cusp.

## 4. Conclusion

The newer technique recommended here allows comprehensive and conservative management of Talon's cusp in a stepwise calculated manner preserving the vitality of the tooth and avoiding patient discomfort.
